# Deep learning reveals disease-specific signatures of white matter pathology in tauopathies

**DOI:** 10.1186/s40478-021-01271-x

**Published:** 2021-10-21

**Authors:** Anthony R. Vega, Rati Chkheidze, Vipul Jarmale, Ping Shang, Chan Foong, Marc I. Diamond, Charles L. White, Satwik Rajaram

**Affiliations:** 1 Lyda Hill Department of Bioinformatics, The University of Texas Southwestern Medical Center, Dallas, USA; 2 Department of Pathology, The University of Texas Southwestern Medical Center, Dallas, USA; 3 Department of Pathology, University of Alabama at Birmingham, Birmingham, USA; 4 Department of Neurology, The University of Texas Southwestern Medical Center, Dallas, USA; 5 Center for Alzheimer’s and Neurodegenerative Diseases, The University of Texas Southwestern Medical Center, Dallas, USA; 6 Peter O’Donnell Jr. Brain Institute, The University of Texas Southwestern Medical Center, Dallas, USA

**Keywords:** Tauopathy, Machine learning, White matter, Histopathology

## Abstract

Although pathology of tauopathies is characterized by abnormal tau protein aggregation in both gray and white matter regions of the brain, neuropathological investigations have generally focused on abnormalities in the cerebral cortex because the canonical aggregates that form the diagnostic criteria for these disorders predominate there. This corticocentric focus tends to deemphasize the relevance of the more complex white matter pathologies, which remain less well characterized and understood. We took a data-driven machine-learning approach to identify novel disease-specific morphologic signatures of white matter aggregates in three tauopathies: Alzheimer disease (AD), progressive supranuclear palsy (PSP), and corticobasal degeneration (CBD). We developed automated approaches using whole slide images of tau immunostained sections from 49 human autopsy brains (16 AD,13 CBD, 20 PSP) to identify cortex/white matter regions and individual tau aggregates, and compared tau-aggregate morphology across these diseases. Tau burden in the gray and white matter for individual subjects strongly correlated in a highly disease-specific fashion. We discovered previously unrecognized tau morphologies for AD, CBD and PSP that may be of importance in disease classification. Intriguingly, our models classified diseases equally well based on either white or gray matter tau staining. Our results suggest that tau pathology in white matter is informative, disease-specific, and linked to gray matter pathology. Machine learning has the potential to reveal latent information in histologic images that may represent previously unrecognized patterns of neuropathology, and additional studies of tau pathology in white matter could improve diagnostic accuracy.

**Supplementary Information:**

The online version contains supplementary material available at 10.1186/s40478-021-01271-x.

## Introduction

Tauopathies are a large and heterogeneous subset of neurodegenerative disorders that include Alzheimer disease (AD), progressive supranuclear palsy (PSP), and corticobasal degeneration (CBD) [24, 34]. They are characterized by accumulation of phosphorylated tau protein. In all three diseases, neuropathological evaluations and neuroimaging studies have indicated widespread changes in tau in both the cerebral cortex (CTX) and white matter (WM). Yet such studies have generally focused on tau aggregation in the cortex, mainly due to the higher prevalence of disease-specific canonical tau aggregates, and higher overall tau burden in some tauopathies (e.g., AD, PSP). While neuropathologic diagnostic criteria for tauopathies are mainly based on cortical aggregates with recognizable phenotypes (neurofibrillary tangles, neuritic plaques, tufted astrocytes, astrocytic plaques, etc.) [4, 5, 10], even these may represent only a small subset of potentially informative pathologic structures. In contrast, white matter pathology in most tauopathies typically lacks similarly recognizable individual aggregates, and consequently is much less well characterized [11, 12, 15, 23, 37]. However, white matter involvement likely contributes to clinical presentation and progression of these disorders. Thus, there is a pressing need for unbiased data-driven approaches to understand its pathophysiologic significance.

White matter pathology in tauopathies is abundant and complex. Hence it is extremely difficult to manually quantify neuropathologic changes, integrate them over slides that may contain hundreds of thousands of aggregates, and then identify aspects that are disease relevant. Visual inspection also has intrinsic human bias, underlying potential inconsistency in diagnosis. On the other hand, automated digital image analysis is ideally suited to reproducibly performing such intricate and repetitive tasks. As a result of recent advances in machine learning, specifically the emergence of deep learning (DL), automated approaches have improved classification accuracies across a variety of bio-image domains [3, 7, 13, 25] and can more readily scale up to larger data sets. Machine learning techniques have recently been adopted for neuropathological applications and demonstrated their ability to recognize pathological aggregates with high accuracy [20, 32, 33]. These approaches have largely focused on recognizing canonical aggregates previously defined based on human observation, and mainly localized in the gray matter.

**Fig. 1:**
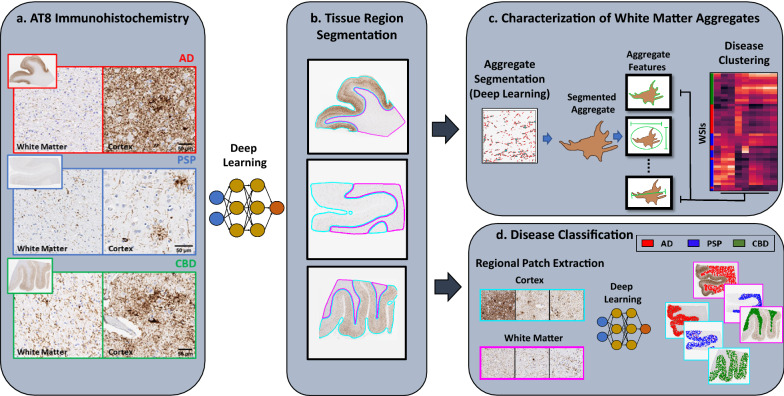
Schematic of analysis workflow. **a** Example images of AT8-stained WSI from AD, PSP, and CBD patients that form the basis of our analysis. **b** Pathologist annotation of cortex(cyan), white matter (magenta) and background (no color) regions were used to train a deep-learning model to segment these regions in WSI. **c** Characterization of white matter aggregates: A pathologist trained deep learning model was used to segment aggregates in the white matter, and for each aggregate, multiple features characterizing its size and shape were extracted. We then performed unsupervised analyses to test whether white matter aggregates in WSI from the same disease were more similar than those from different diseases. **d** Disease classification based on cortex and white matter: Separate deep learning models were trained for the cortex and white matter to predict disease status directly from image patches (without need for any human curation of features) and the performance of these two models was compared and contrasted

Consequently, we developed deep learning approaches to better understand the role of white matter tau aggregates in AD, PSP and CBD by analyzing AT8 stained whole slide images (WSI). We created algorithms to (a) demarcate white matter regions (Fig. 1b); (b) identify tau aggregates and characterize their changes across diseases (Fig. 1c); and (c) compare the disease classification of deep learning models trained on gray and white matter pathology (Fig. 1d). Our results indicate a strong, and disease-specific, relationship between tau burden in the gray and white matter, suggesting a common pathophysiological origin. Using unsupervised approaches, we determined that WM tau aggregates in AD, CBD and PSP are highly distinct, and we have identified disease-specific features of aggregate morphology. Finally, we found that these tauopathies could be equivalently classified based on white or gray matter tau staining, underscoring the importance of white matter pathology. Thus, machine learning has enormous potential to reveal unrecognized patterns in complex neuropathological images, and may help further classify tauopathies.

## Methods

### Neuropathologic evaluation, immunohistochemistry and whole slide scanning

Forty-nine autopsy brain cases were retrieved from the UT Southwestern Neuropathology archives, including 16 cases diagnosed as AD, 13 cases as CBD, and 20 cases as PSP (Additional file 1: Table S1). Involvement of the frontal cortex and white matter is associated with advanced disease for all three tauopathies, and therefore the presence of tau aggregates in the frontal gray and white matter was the main inclusion criterion. All AD cases featured Braak neurofibrillary stages V and VI, and all CBD and PSP cases featured some degree of frontal cortical and white matter involvement. Since the major focus of this study was the tau pathology in the neocortex and subjacent white matter, CBD and PSP cases with coexistent low levels of Alzheimer type neuropathological change (Braak neurofibrillary stages I and II, where tau pathology is essentially restricted to the entorhinal cortex) were not excluded.

All sections were cut from formalin fixed, paraffin embedded tissue blocks at 5 micron thickness and stained with anti-AT8 antibody (Thermo Scientific MN1020, 1:200 dilution) using Leica Bond III automated immunostaining platform. Monoclonal antibody AT8, first reported in 1992 by Mercken et al. [28], was developed against phosphorylated tau isolated from human Alzheimer disease brain. We selected this anti-phospho-tau antibody because it has been used in laboratories worldwide for decades for the immunohistochemical detection of disease-associated phospho-tau in many different neurodegenerative conditions. In 2003, Arai et al. [1] investigated the differences in immunoreactivity of 13 antibodies to epitopes spanning the entire length of the tau molecule to phospho-tau lesions in autopsy brain tissue from subjects with Alzheimer disease, Pick disease, progressive supranuclear palsy, and corticobasal degeneration. While antibodies directed at epitopes within the microtubule binding domain of tau showed different levels of immunoreactivity among these tauopathies, antibodies to the middle region of tau, including AT8, showed similar immunoreactivity among all 4 tauopathies. Importantly, we also included exposure of tissue sections to concentrated formic acid preceding epitope retrieval and immunostaining, as this has been shown to maximize the specific immunoreactivity of tau lesions for use in quantitative image analysis applications [8].

AT8 stained slides were digitized using an Aperio ScanScope CS2 (Leica Biosystems, Buffalo Grove, Il) whole slide scanner at 20X magnification.

We note that only frontal regions were used for the analysis of cortical and white matter tau aggregation patterns. However, to provide additional data to improve the accuracy of our classifier that distinguishes cortex from white matter, we additionally incorporated hippocampal, temporal, parietal and occipital regions from each case. All DL models described below were trained using either a Tesla p40 or v100 single Graphical Processing Units with the open-source package TensorFlow.

### Model training for region (cortex vs white matter) identification

#### Data

For region identification (as opposed to disease/aggregate analysis) a total of 37 WSIs (16 AD, 11 PSP, 10 CBD) imaged at 20X magnification (0.5 microns per pixel) were used for training and testing. Among these WSIs, 21 were taken from the middle frontal gyrus, the focus of our study, while the remaining were taken from the hippocampus, superior temporal gyrus, lateral parietal cortex, and calcarine cortex. WSIs were first annotated by a trained neuropathologist, using the QuPath bioimage analysis software [2], to indicate cortex and white matter regions, as well as background areas. From each annotated WSI, we generated an average of ≈ 12,000 image patches (128 × 128 microns = 256 × 256 pixel at 20× magnification) giving a total of ≈ 430,000 patches to be used as input for the DL model. WSIs (and correspondingly the patches derived from them) were split using threefold cross validation, with random assignments of diseases to folds while ensuring that each fold contained the same proportions of slides from each disease.

#### Model

We used a fully convolutional network (FCN) with a custom architecture (Additional file 1: Table S2), which takes as input an image patch and predicts whether the center pixel of the patch was in the cortex, white matter or background. We trained a different model for each fold, giving us three models in all.

#### Training

During training, images were transformed with the following augmentations: random horizontal flip, random vertical flip, and random addition to hue and saturation from the Imgaug library (https://github.com/aleju/imgaug). We used a categorical cross entropy loss, with different weights for each class based on their frequency in data to account for class imbalance. We used an Adam optimizer with a learning rate of 0.0001. Model training was performed for 20 epochs, after which each model achieved an average classification accuracy of at least 92% on training data and 85% on testing data.

#### Evaluation

For the final region identification, we applied all three models at the whole slide level in fully convolutional fashion [26] and took their consensus prediction to achieve the most accurate result. Specifically, for each model we took their values at the activation layer (i.e., class response for WM/CTX/Background), averaged them together across models, and then selected the class with the the maximum average response as the final classification. Lastly, post-processing steps using the python library, scikit-learn, functions remove_small_objects and remove_small holes (area threshold of 1000 pixels) were applied to further refine region identification. Consensus predictions were then evaluated by pathologists.

### Model training for aggregate identification

#### Data

A total of 18 WSIs (6 AD, 6 PSP, 6 CBD) taken from the middle frontal gyrus and imaged at 20X magnification (0.5 microns per pixel) were used for training and testing. Aggregates were identified under pathologist supervision using the QuPath software as follows. First, to standardize staining levels across slides, tissue samples were pre-processed using the Estimate Stain Vectors function in QuPath [2]. Next, using the rectangle tool, 4–6 regions of interest (ROIs) were next selected across the white matter areas of the sample. Finally, manual thresholding of the DAB signal was performed using the positive pixel count algorithm to identify AT8 positive areas. These areas were manually inspected and then exported to a format readable by our machine learning pipelines, such that each pixel in the analyzed rectangular area was identified as belonging to an aggregate (positive DAB staining), background (negative brown staining) or edge (at the border of aggregates and background) to serve as ground truth for training our models. In all, 74 rectangular regions with sizes between 160 K and 4 M microns^2^ (~ 1 to 17 M pixels) were generated. For 3 out of the 18 WSIs (one from each disease) we additionally generated ROIs from the cortex in the same manner to test the ability of our models to detect tau aggregates in this region.

#### Model

To get single pixel resolution in our aggregate prediction we employed a convolutional neural network with UNet architecture [30]. The model takes as input immunohistochemistry (IHC) image patches and outputs patches of the same size so as to match the pathologist-generated ground truth where each pixel is classified as aggregate, background or edge. WSIs were split using twofold cross validation, such that each fold had equal number of slides from each disease. We trained a separate model for each fold.

#### Training

During training, images were transformed with the following augmentations from the Imgaug library: random horizontal flip, random vertical flip, and random addition to hue and saturation. The models were trained on 400 × 400 pixel tiles randomly sampled the rectangular regions identified above. We used a categorical class entropy loss (measuring difference between the predicted and true pixel classes) with different weights for each class based on their frequency in data to account for class imbalance. Additionally, to prevent over- or under-splitting of aggregates we added an additional image level loss that encourages the prediction to produce the same overall amount of edges as the ground truth. We used a Stochastic Gradient Descent optimizer with a learning rate of 0.001 and momentum of 0.5. Model training was performed for 50 epochs, after which our model achieved an average classification accuracy of at least 89% on training data and 82% on testing data.

#### Evaluation

From our two trained models, we selected the one with a higher cross-validation accuracy. For each WSI, we then applied our aggregate classifier across the entire slide, to generate an output image “mask” of the same size as the input slide with each pixel classified as background, aggregate or edge. During classification, we implemented a response threshold of 0.5, such that aggregate and edge predictions with a lower class response (i.e., low classification confidence) were classified as background instead. WSIs and their aggregate masks were evaluated side-by-side by pathologists.

#### Calculation of tau burden

We used output region masks from our region classifier to calculate the total area of a region, either cortex or white matter, in a WSI. We then quantified the tau burden of that region in terms of the fraction of area occupied by aggregates (i.e., pixels classified as Aggregate by the aggregate classifier).

### Hand-crafted feature analysis

#### Aggregate identification

From our aggregate masks, as described above, we first identified individual aggregates in the white matter region (as determined by the region classifier). Specifically, aggregates were identified by locating connected island of pixels classified as aggregate class, with distinct aggregates separated by pixels classified as either background or edge.

#### Aggregate features

For each individual aggregate, we then used in-house software to calculate our set of hand-crafted features (Additional file 1: Table S3).

#### Aggregate quality control

Based on the inspection of a pathologist, we observed two types of artifacts in aggregate objects. The first was objects that were deemed too small to be considered an aggregate and were removed using the sklearn function remove_small_objects with an area minimum threshold of 30 pixels. The second type of artifact was rarer instances of AT8 signal localized within nuclei but not attributable to a mature aggregate. To remove these artifacts from further analysis, we first tested whether our handcrafted features with the addition of texture features (Additional file 1: Table S4) recognized them. To this end, we took a subset of 200 aggregates from each WSI, extracted features for each and clustered them using Uniform Manifold Approximation and Projection(UMAP) [27]. After verifying that these artifacts formed a unique cluster apart from other aggregates, we trained a random forest classifier with these data to classify objects as either an aggregate or an artifact, and tested performance on a held out portion of the data. For all subsequent analysis, we applied the trained random forest model to filter out these artifacts.

#### Slide-level analysis

After removing the artifacts (~ 4% of all initially detected aggregates) that failed our quality control procedure, for each feature, we calculated its median value across all aggregates in the white matter region of each WSI. We performed unsupervised clustering of WSIs based on these median feature values using the Clustermap function from the Seaborn toolbox with the metric ‘Correlation’ and average linkage. For each feature, we used a Mann–Whitney test, with Bonferroni-based multiple-hypothesis testing across disease comparison, to test whether feature values differed across the 3 diseases.

### Automated disease classification using deep learning

We trained separate DL models on the cortex and white matter to predict disease (AD/CBD/PSP) directly from AT8 stained images (i.e., relevant image features are learned by the model without any need for manual curation of features as above).

#### Data

A total of 49 WSIs (16 AD, 20 PSP, 13 CBD) taken from the middle frontal gyrus and imaged at 20X magnification (0.5 microns per pixel) were used for training and testing. From each WSI, we generated 10,000 image patches (224 × 224 pixel, 112 × 112 micron) from either the cortex or white matter regions to be used as input for the DL model.

#### Model

For our DL model, we used a multiple instance learning (MIL) network in order to mitigate the influence of uninformative patches on model training [17]. An MIL framework makes predictions on a group, or batch, of patches rather than an individual patch (Additional file 1: Figure S1), thereby better accommodating patches which lack any disease-specific information (e.g., those without aggregates).

#### Training

WSIs were split using threefold cross validation, keeping the distribution of data from each disease consistent across each fold. We trained two models for each fold, one using data from the cortex regions of the WSIs, and one using data from the white matter regions, to assign AT8 stained image patches to their corresponding diseases. We used a binary cross-entropy bag loss with different weights for each class based on their frequency in data to account for with class imbalance. We used an Stochastic Gradient Descent optimizer with a learning rate of 0.0001 and a momentum of 0.5. We trained each model for 3 epochs, after which our cortex and white matter models achieved an average patch-level classification accuracy of at least 93% and 90% on testing data, respectively.

#### Evaluation

Each of the 3 cross-validation models was applied solely to data in its corresponding testing set (i.e., had not been used in training). For validation, 1000 patches were extracted from each slide. Individual patches were classified into the 3 diseases to measure patch level performance. To determine disease classification at the slide level, we calculated the disease class as the majority class from those 1000 patches.

### Interpretation of deep learning classification features

#### UMAP clustering

We used 2000 image patches from the testing set of a single fold from our Disease Classification DL model. From each image patch, we extracted 1024 features using the second to last layer of our DL model (i.e., layer before disease classification). We then used the output feature matrix (2000 × 1024) as input for the UMAP function with an n_neighbors value of 5 and a min_dist of 1.

#### Handcrafted features calculation

For each image patch described above, we applied our aggregate classifier to segment aggregates and removed artifacts as described in the Aggregate Quality Control section. We next calculated the feature values for Area, Eccentricity, and Minor Axis length as described in Aggregate Features section and represented each patch by the mean feature values of the aggregates they contained.

## Results

### Automated identification of white matter and aggregates

**Fig. 2:**
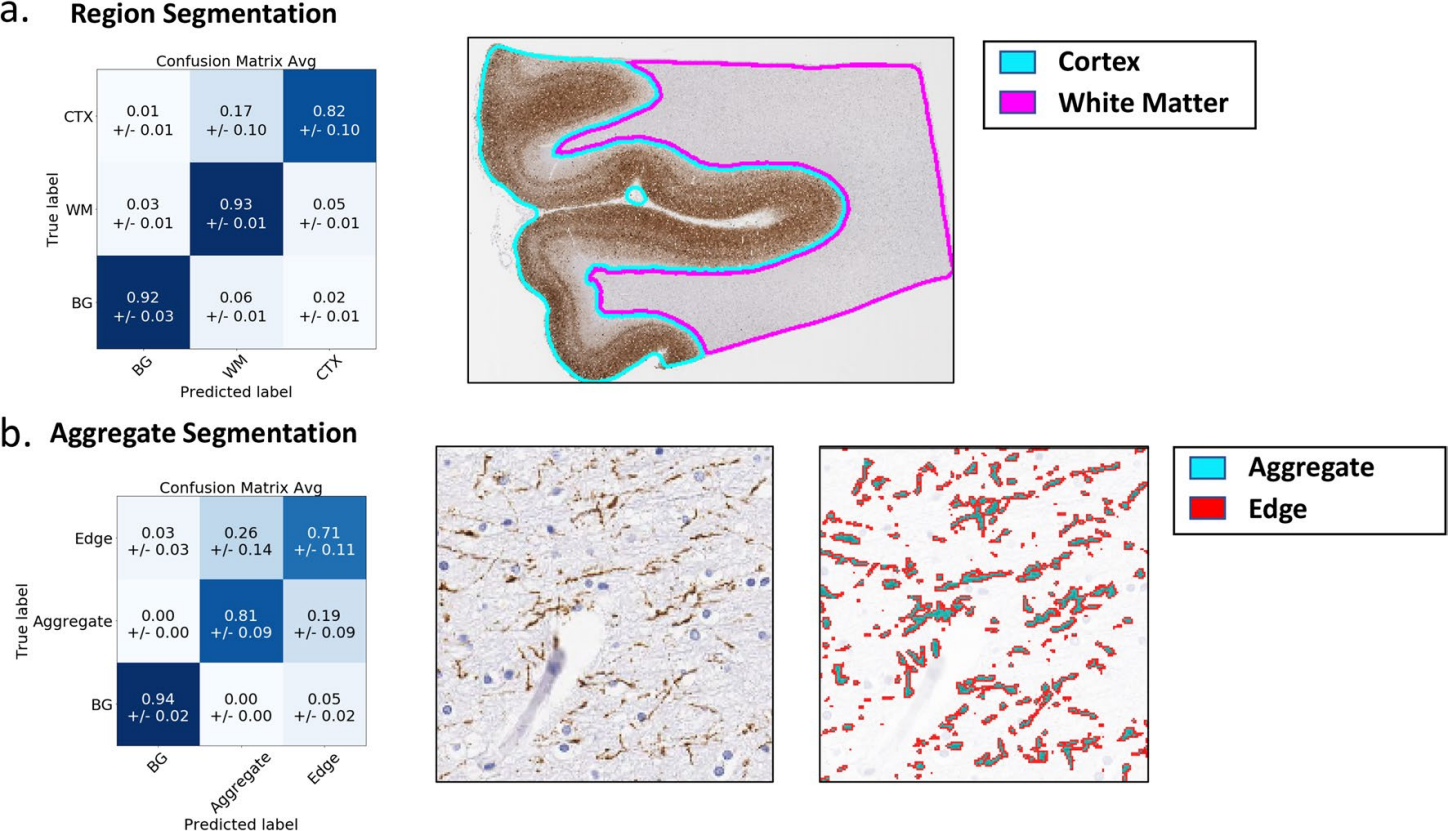
DL accurately identifies tissue regions and AT8-stained aggregates. For both region and aggregate models, a cross-validation scheme was used to generate multiple models, each trained on part of the data and evaluated on the rest. We report performance (left column) using confusion matrices which show how image areas with known true labels (rows) are assigned to different predicted classes (columns). Values denote fractions of patches/aggregates belonging to true class (i.e., rows add up to one) assigned to corresponding predicted class averaged across cross-validation models, with standard deviations shown in parenthesis. Right column shows sample classification results. Note: BG = Background. **a** Region segmentation: threefold cross validation was used and performance is measured in terms of fraction of image-patches from a region that are correctly classified (diagonal entries in dark blue). **b** Aggregate segmentation: twofold cross validation was used and performance is measured in terms of fraction of pixels that are correctly classified

We first sought to automate the process of (1) segmenting gross cortex and WM areas; and (2) identifying individual tau aggregates. While manual annotations by trained pathologists could be used for these tasks, the number of annotations required would be both laborious and time-consuming. Therefore, we instead automated these tasks by training two separate deep learning (DL) models from a significantly smaller amount of pathologist annotation data and applying them to our full dataset (Fig. 2).

We first trained DL models to segment tissue regions as cortex, WM or background, based on pathologist demarcations of these regions in 37 AT8 stained WSI. Of these WSI, 21 are from the middle frontal gyrus, which is the focus of this study, while the remaining are from the hippocampus, superior temporal gyrus, lateral parietal cortex, and calcarine cortex. To test how our models generalized to images on which they were not trained, we adopted a threefold cross validation approach: we split the slides into three groups, trained 3 models (each trained on 2 of the three parts), and finally tested the performance of each model on the third of slides it had not seen (“Methods”). Our model accurately classified cortex, WM and background image patches 89% of the time (Fig. 2a). This ability to distinguish WM and cortex was true for all three diseases, although our model struggled most with CBD cases where tau burden was comparable in white matter and cortex (Additional file 1: Figure S2a). Satisfied with the performance of these models, for further analysis we employed a consensus model that considers the average prediction from all three models (“Methods”). We used this consensus model to profile all 49 middle-frontal gyrus WSI used in this study, and two independent neuropathologists (RC and CW) confirmed the accurate segmentation of the WM and cortex regions in these slides (Additional file 1: Figure S2b).

We similarly trained a model to identify individual aggregates based on pathologist annotations of aggregate boundaries (approximate 360,000 annotated aggregates) in 74 WM regions from 18 AT8 stained images of the middle-frontal gyrus region. This model classifies each pixel in an image as background (no AT8 staining), aggregate (pixels with AT8 staining) or edge (at the border of an aggregate). The explicit inclusion of the edge class encourages our model to accurately capture aggregate boundaries (e.g., by separating neighboring aggregates) which is important for the downstream analysis of aggregate shape. We trained the model using 9 of the 18 slides and tested performance on the 9 remaining slides (three from each disease). The model distinguished aggregates from background with high accuracy, as shown by our pixel metrics, however the boundary between edge and aggregate pixels was sometimes confused (i.e., the single pixel boundary between these classes would shift toward or away from the aggregate) (Fig. 2b, Additional file 1: Figure S3a). We found that this minor confusion did not affect our ability to measure aggregate shape, as evidenced by the segmentation metrics and strong correlation between extracted downstream features in true and predicted aggregates (Additional file 1: Figure S4). We also tested the performance of this model (trained in WM) on cortical regions. While the identification of AT8 staining vs background was accurate, in areas where cortical density of aggregates was high, the model (or even trained pathologists) could not always separate individual aggregates (Additional file 1: Figure S3b). Thus, we felt confident in using this model for identification of total tau staining in cortex and for identification of individual aggregates in the WM.

### Quantification of tau burden

**Fig. 3:**
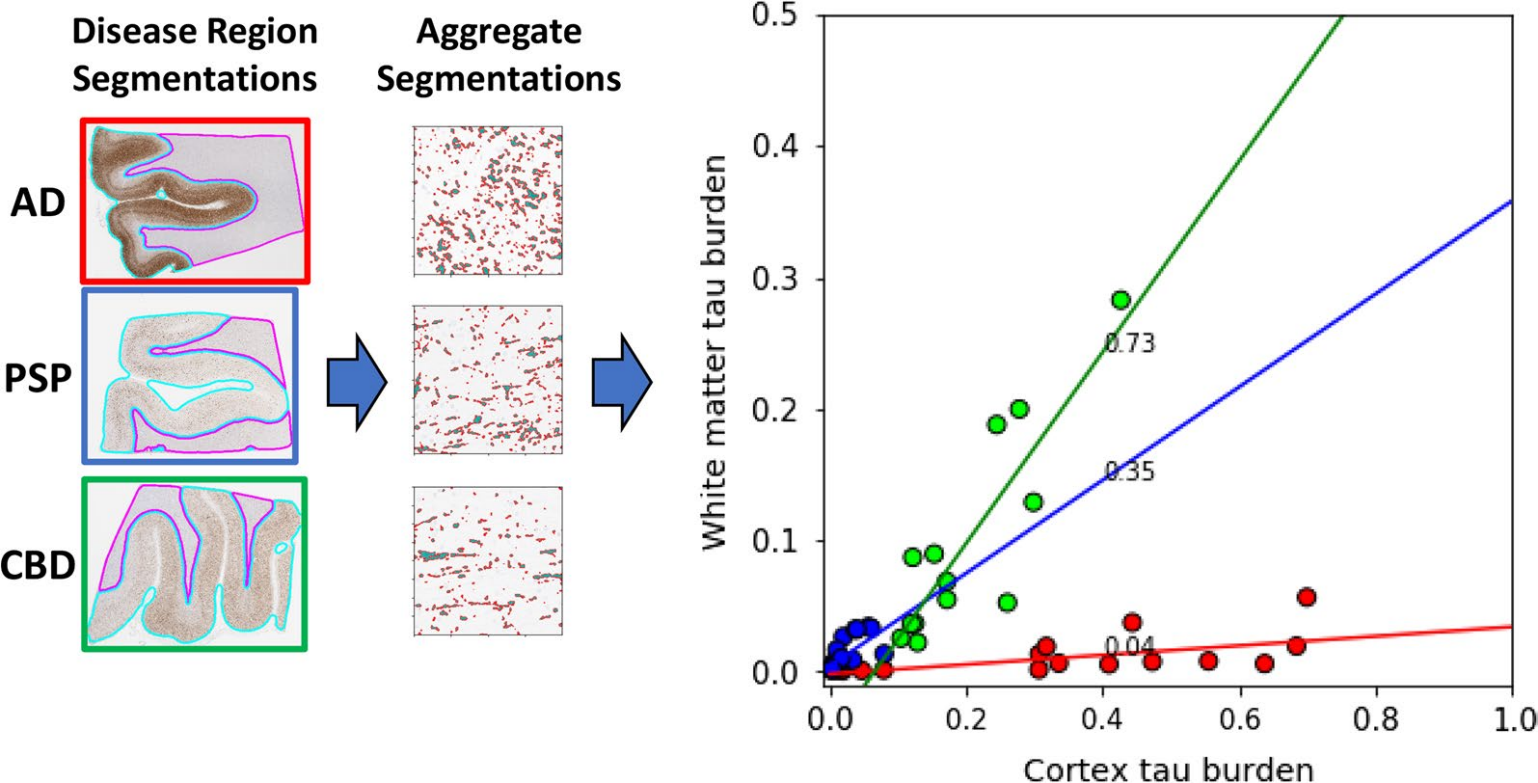
Tau burden is disease specific. Scatter plot of tau burden in cortex (x-axis) vs WM (y-axis) regions across multiple WSI (individual data points) from different diseases (point colors). Tau burden in a region was estimated by the ratio of area covered by tau aggregates (from aggregate classifier) to total area of the region (WM/cortex from the region classifier). Data from each disease are fit using a least squares straight-line fit and the best-fit slope is shown next to the fitted line

While it is known that tau aggregates are present in both WM and cortex, it is unclear how these regions relate, and whether any relationship is disease-specific. To explore this, for each sample we calculated the fraction of area occupied by tau aggregates (as determined by our aggregate model), a proxy for tau burden, in the WM and cortex regions. For a given disease, we observed a strong linear relationship between tau burden in the WM and cortex: samples with a higher tau burden in the cortex also showed increased tau burden in the WM (Fig. 3). Interestingly, the distribution of tau burden between the cortex and WM appeared disease-specific. AD samples largely displayed high tau burden in the cortex, but very little in the WM (an order of magnitude less than the cortex). Only a small subset of AD samples appeared to deviate from these trends, exhibiting unusually low tau burden in the cortex. In contrast, in both PSP and CBD the tau burden in the WM and cortex were more comparable (burden in WM was 1/3rd of the cortex in PSP and 3/4th in CBD), although CBD samples frequently displayed a higher burden than PSP in both regions. Congruent with this finding, a recent study also found that different tauopathies also displayed distinct tau burden in the white matter [16]. These results indicate that consideration of tau burden in both the cortex and WM could lead to improved disease separation, and prompted us to further examine properties of aggregates in the WM.

### Morphological characterization of tau aggregates

**Fig. 4:**
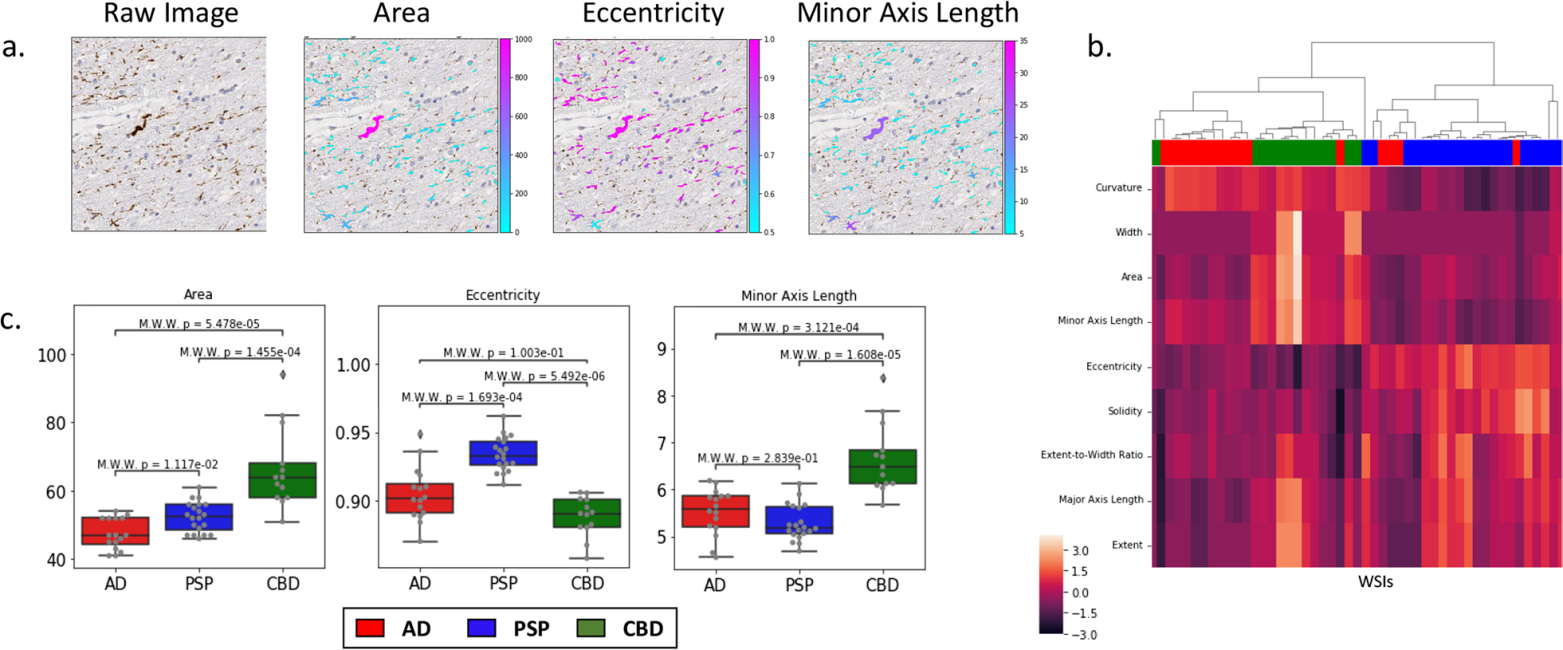
Aggregates from different diseases show distinct shapes and sizes. Individual aggregates were identified in the WM of WSI and were characterized by features describing their size and shape. **a** Example image patch (left) from a WSI with individual aggregates colored based on sample features: area, eccentricity, and minor axis length. **b** Individual WSI (rows) were characterized based on median feature values (columns) of aggregates in their WM and were ordered based on hierarchical clustering. Values within each feature were z-score normalized to allow comparison across features. Colors on top (Red/blue/green) indicate disease associated with each WSI. **c** Boxplots of median feature values for area, eccentricity, and minor axis length shown in WM regions of WSI (gray dots) compared across each disease. Mann–Whitney test, with Bonferroni-based multiple-hypothesis testing correction (across diseases) was used for statistical comparison

We sought to understand how aggregate morphology in the WM differed in AD, PSP and CBD. Unlike in the cortex, where tau aggregates in different diseases differ in the occurrence of stereotypical morphologies (e.g., neurofibrillary tangles and tufted astrocytes), less is known about differences between the aggregates in the WM among these disorders. We therefore profiled aggregate morphology based on a panel of features capturing different aspects of size (i.e., area, length, width) and shape (i.e., eccentricity, curvature, solidity) (Fig. 4a, Additional file 1: Table S3). We excluded features such as texture or staining intensity, as these are harder to interpret and more susceptible to experimental variability. Additionally, we removed false aggregate detections arising from rare off-target staining (“Methods”) although the overall results are largely unaffected by this process (Additional file 1: Figure S5). For each WSI, we then took the median value of each morphological feature across all aggregates in the WM to generate a representative WM morphological profile of that slide.

To test, in an unsupervised fashion, the relationship between disease and aggregate morphology we performed hierarchical clustering of the WM morphological profiles (Fig. 4b). Surprisingly, despite the general-purpose collection of shape features present in our morphological profiles, we saw definite clustering among WSI from the same disease. The aggregate data from CBD WSIs stood out because of their larger values of features characterizing aggregate size (e.g., area). Aggregates from PSP WSIs exhibited similar length (major axis length and extent) as CBD, but based on their greater eccentricity and lower values of minor axis length, width and curvature, seemed to be long and linear. In contrast, AD aggregates had shorter lengths but showed increased curvature suggestive of short curled segments. Finally, closer inspection of the AD WSIs that did not cluster well revealed (Additional file 1: Figure S6) that these WSIs were also outliers in our tau burden analysis that had unusually low tau burden in WM regions, possibly making them more susceptible to fluctuations and the occasional segmentation error.

To determine whether these observed differences in median feature values were statistically significant, we next compared their distributions between diseases (Fig. 4c). Of the 9 features examined, 3 showed statistically significant differences for CBD compared to the other tauopathies, while 2 showed statistically significant differences for PSP compared to the other tauopathies (Additional file 1: Figure S7). Among the most prominent differences, we found that AD and PSP aggregates had reduced area and thickness than CBD, while in AD aggregates were significantly shorter (extent and major axis length) than in the others. These statistical results are consistent with a qualitative characterization of aggregates in CBD being large and round, those in PSP being long, thin, and straight, and AD exhibiting short curly aggregates.

### Disease classification of tauopathies using either cortex of white matter pathology

**Fig. 5:**
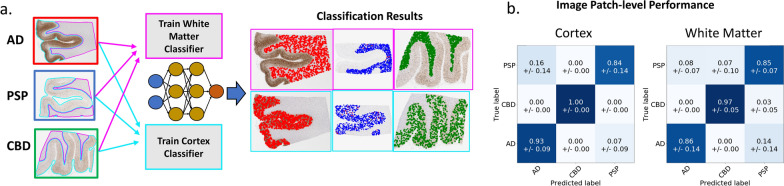
Disease classification from DL. **a** Overview of our DL approach in which cortex/WM regions are used to train two separate DL models for disease classification at an image patch level. **b** Confusion matrices comparing the average patch-level classification accuracy using cortex (left) or WM (right) data

While our results show that WM aggregate morphologies differ on average between diseases, we sought to determine whether (a) the phenotypic differences were strong enough to allow disease classification based on localized tissue patches, and (b) how discriminative power based on WM compared with using cortex pathological information (Fig. 5a). We opted to use a deep learning strategy over hand-crafted features because: (1) segmentation accuracy of individual aggregates in the cortex dropped when pathology became exceedingly dense, which might bias performance measurements when comparing cortex/WM classification; and (2) a DL approach enabled our analysis to go beyond our selected hand-crafted features and utilize all available information from tissue data. To this end, we trained two separate DL classifiers on tissue data solely from either (1) cortex region segmentations or (2) WM region segmentations. To ensure fairness in comparisons, both were trained and tested on the same WSIs using three-fold cross-validation, resulting in 3 trained models per region. We first evaluated classifier performance by comparing their accuracy on individual image patches from each testing set. Strikingly, both models achieved a high accuracy (greater than 90%) with our cortex model performing only slightly better on AD samples (Fig. 5b). The high accuracy obtained by the WM DL classifier reinforces our previous findings that pathology in these regions contain disease-specific signal and suggests that their specificity may be nearly as distinct as that in the cortex.

We next sought to understand whether the WM and cortex-based classifiers struggled on the same samples or whether these two regions provided complementary information for disease classification. As above, we analyzed patches from our full panel of 49 WSI, and quantified what fraction of patches in each sample were assigned to the correct disease type by the 2 models. The majority of WSIs (43) were accurately classified (> 50%) by both DL classifiers (Additional file 1: Figure S8a). Of the remaining 6 that were misclassified, 2 were misclassified by both classifiers, 2 were only misclassified by our WM classifier, and 2 were only misclassified by our cortex classifier. Indeed, by taking the consensus result of both cortex and WM classifiers, we achieved the highest classification accuracy, albeit marginally (Additional file 1: Figure S8b). Together, these results indicate that pathology from the cortex and white matter largely complement each other, and in some cases may offer additive information.

### Relating machine learning features to visual aggregate phenotypes

**Fig. 6:**
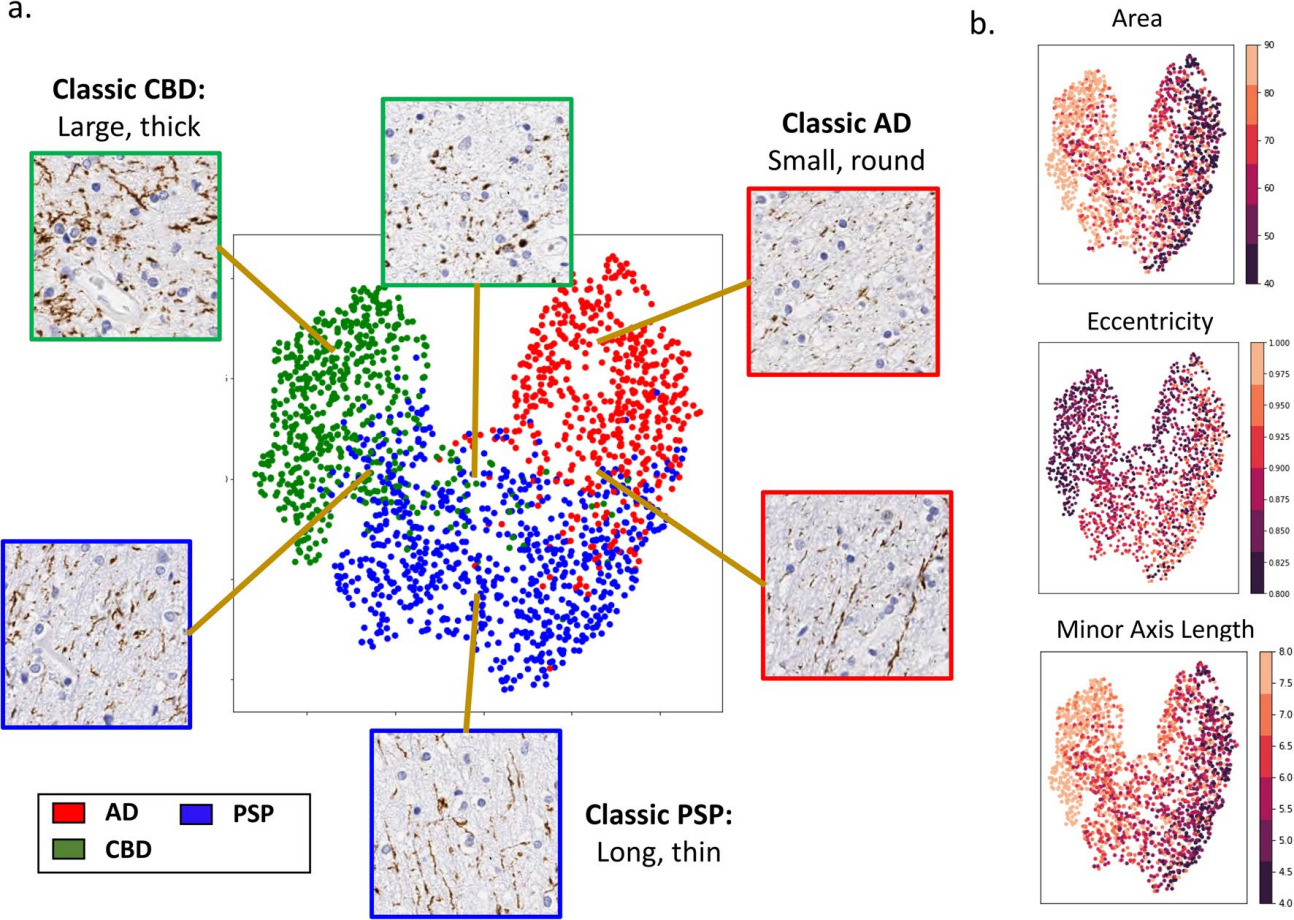
Interpretation of WM pathological features used for disease classification. **a** UMAP visualization of image patches clustered based on their similarity as perceived by the automated disease classifier (based on the 1024-dimensional output of the penultimate layer of the model; “Methods”). Data points are colored based on their ground truth disease label and representative images of data at different areas within clusters are displayed. **b** The same UMAP visualization from **a** with data points instead colored by the average feature values (across aggregates in the corresponding image patch) for area, eccentricity, and minor axis length

Finally, given the black box nature of our automated disease classification and the disconnect between our aggregate features (such as minor-axis length) and descriptions of aggregates in neuropathology, we sought to reconcile these different descriptors. Accordingly, we arranged white matter image patches from AD, PSP and CBD based on their similarity as perceived by the automated disease classifier (Fig. 6a, “Methods”; nearby points represent more similar patches), along with the values of prominent feature descriptors for aggregates in these patches (Fig. 6b). Additionally, we displayed sample image patches from different regions of these plots to help connect our image descriptors to visual phenotypes. As expected, our data formed distinct clusters for each disease (Fig. 6a, AD: red, PSP: blue, CBD: green). Encouragingly, we observed that image data from the center of each cluster reinforce our findings based on the hand-crafted features (Fig. 6a). Specifically, PSP aggregates were long and thin with high eccentricity and low minor axis length, AD aggregates were small and round, with lower eccentricity, and CBD aggregates were bigger and less straight, with larger areas. As we move from disease centers towards neighboring disease clusters, we observe a continuous transition in phenotypes and the associated image descriptors, with aggregates near the border of two clusters exhibiting a mixture of the characteristic morphologies and handcrafted features (highlighting the heterogeneity of aggregates in the white matter). Taken together, these results suggest the consistency between our different approaches and provide a means to connect them to more conventional neuropathological descriptions.

## Discussion

Pathologist characterization of aggregate staining has long been the gold standard for defining neurodegenerative diseases. However, there is potential bias in the development of such diagnostic criteria, be it in terms of the regions analyzed (e.g., gray vs white) due to their perceived disease relevance, or the choice of aggregates characterized (e.g., only a subset of easily recognizable aggregates are used in distinguishing tauopathies). Given the diversity of clinical features within a single diagnosis, a deeper characterization of structural pathology might improve disease stratification and clinico-pathologic correlation. A challenge to developing novel pathologic criteria is the quantity and complexity of aggregate phenotypes within a single WSI. In the current study, we sought to understand the nature and disease-specificity of tau aggregate morphology in the WM across different tauopathies. Thus, we developed a computational pipeline to analyze and quantify tau staining in whole slide images of AT8 stained tissue from patients suffering from AD, PSP, or CBD. We first developed and validated DL models to automatically demarcate the cortex and WM regions in AT8-stained WSIs and identify tau aggregates therein. Then, in contrast to previous efforts that have employed pathologist-derived scores for classification [6, 21], this study uniquely employed cutting edge ML technology to accurately identify every individual tau aggregate in the white matter of AD, PSP, and CBD cases, dissect and quantify morphological features of these aggregates, and identify disease-specific signatures.

Past work has demonstrated tau pathology involves neurons and glia in both gray and white matter compartments, but the involvement of these cellular and anatomic compartments varies amongst the different tauopathies. AD is known to be a mainly neuronal tauopathy, whereas PSP and CBD have a higher fraction of glial involvement [14, 15, 18, 22]. These differences are reflected in the canonical cortical tau aggregates specific for each disorder: neurofibrillary tangles along with neuritic plaques are characteristic for AD, tufted astrocytes for PSP, and astrocytic plaques for CBD. Based on the staining characteristic of these canonical aggregates, it is not hard to determine what cellular compartment they involve (also implied in their names), but most of the time, these canonical aggregates only represent a small fraction of all tau aggregates both in gray and white matter [9, 15, 35]. Tau burden in gray and white matter is also variable across these diseases, and although it is not entirely clear what are the exact roles of gray and white matter involvement in disease progression and propagation, studies in animal models suggest that white matter may be involved in disease propagation to remote sites [29].

### Characterization of white matter pathology

Our work recapitulates the disease specific findings for tau burden such as higher white matter tau pathology in CBD [16]. However, a novel aspect is the simultaneous quantification of cortical and white matter tau burden within an individual subject which we show is highly correlated in disease-specific fashion: tau burden in AD samples was largely localized to the cortex, while CBD and PSP both displayed a more equitable distribution of tau between the two regions, with CBD samples frequently displaying a higher tau burden than PSP in both regions. The strong correlation between WM and cortical burdens at a single patient level for a disease suggests that similar pathophysiologies are at play in these regions. Next, we determined that WM aggregates in AD, CBD and PSP are morphologically distinct. Indeed, the diseases group separately even with a simple unsupervised clustering based on just a few features characterizing size and shape of the aggregates, reinforcing the substantial differences in their phenotypes. This approach of extracting “hand-crafted” features allowed us to infer interpretable disease-specific differences: WM aggregates in CBD are large, and round, those in PSP are long, thin, and straight, and those in AD are short and curly. Lastly, we built a DL pipeline to distinguish AD, CBD and PSP based on either their WM or cortical tau staining (using all aggregate properties, not just size and shape). The performance of classification based on WM and cortex are comparable, and can be complementary (combining both regions improved performance). In addition to more accurate disease classification, combining both regions may provide better insight into the pathophysiology of the variation of cortical and WM tau distributions within the spectrum of each tauopathy, as well as across different tauopathies.

### Future work and limitations of current study

While our studies reveal clear disease-specific morphological differences in tau aggregates, they do not yet explain the underlying cell-biological reasons for these differences. For canonical cortical aggregates (e.g., neurofibrillary tangles or tufted astrocytes) in various tauopathies, their shape has been used to infer the cellular compartment where the tau aggregate resides. The lack of characteristic aggregate morphologies has prevented a similar approach in the WM. We believe our ability to quantify disease specific aggregate phenotypes, identify which ones are most disease specific, and relate them back to the original images, makes it possible to generate testable hypotheses in this regard. For example, high eccentricity and short minor axis length seen in PSP, which is suggestive of long, narrow and continuous aggregates, is consistent with axonal projections. Such hypotheses can drive future studies that will help us understand how cell type and subcellular compartment impact aggregate morphology in the WM. Another powerful future application of our work is to help discriminate rare variants that are hard to distinguish from the diseases studied here, for example FTLD-MAPT-NOS cases or cases that are difficult to discriminate as either PSP or CBD. Finally, our approaches are very general and could be easily extended beyond the scope of tauopathies to study the role of WM pathology in other neurodegenerative diseases.

We note however that some degree of caution must be exercised in interpreting our results. First, the cases selected may not reflect the full range of heterogeneity in these diseases. It will be important to capture additional sources of biological and technical variation by testing these approaches in a larger independent data set spanning multiple institutions. Similarly, for this proof of principle study we restricted ourselves to a single brain region (frontal cortex and WM) with definitive ground truth disease assignments. Future studies can test the robustness of our results in other brain regions and samples that contain co-morbidities. Additionally, we note that the current study was restricted to the AT8 antibody. While morphology of AT8 aggregates is widely used for the diagnosis of AD, PSP, and CBD and AT8 is highly sensitive to phosphorylated tau protein, it is not specific to either 3R and 4R phosphorylated tau protein whose presence varies across these diseases [23]. Thus, further insights may be gained by the use of 3R and 4R specific antibodies. Finally, an inherent limitation of studying WSIs is the inability to capture the 3D structure of objects present in brain tissue. Axonal projections, for example, might appear either elongated or punctate when viewed from a two-dimensional slide depending on their orientation during tissue sectioning. Here, by averaging results across a slide we hoped to capture a range of 3D orientations, and our results suggest that this was at least partly successful. Nonetheless, it would be undoubtedly more powerful to take into account aggregate and slide orientation or to perform a full 3D analysis.

### Machine learning applications in neuropathology

While the present work focuses on existing disease definitions, it also highlights a far larger opportunity for machine learning to aid in the development of new, more clinically prognostic, stratifications of disease. Each tauopathy itself has a spectrum of clinical and radiographic presentations [23], and applying our current approach to study morphological differences between tau aggregates within each disease or commonalities across different tauopathies may help us to identify disease subtypes. These stratifications could then be correlated with or refined by clinical outcomes. Furthermore, it is known from biochemical studies [19, 31, 36], and more recently cryo-EM studies that different protein strains are present in tauopathies and other neurodegenerative diseases (e.g., synucleinopathies). Thus, a key question, as we continue to obtain structural insights from these strain differences, is how structural similarity at the protein level maps to morphological similarity at the aggregate level and how these relations predict disease spread within the brain.

In summary, we demonstrate how deep learning approaches can be used to characterize tau aggregation in the WM of WSI from AD, PSP and CBD patients. WM aggregates are relatively less studied than those in the cortex and do not necessarily display the stereotypical morphologies (e.g., plaques, tangles) seen in the cortex. Yet by integrating image features across thousands of aggregates, our ML approaches identified characteristic disease-specific differences in WM aggregate morphology, with discriminative power comparable to that from analysis of cortex. Our results specifically highlight the need of further studying tau aggregation in the WM and more broadly the value of ML driven studies in the field of neuropathology.

## Supplementary Information

**Additional file 1.** This file contains supplementary figures S1–S8 and tables S1–S4 describing data and additional analyses related to the study.

## Data Availability

The datasets analyzed during the current study available from the corresponding author on reasonable request. Code used in this work is available at https://github.com/Rajaram-Lab/anc-2021-dl-wm-tauopathy.
