# Supplementary material for: Deep learning reveals disease-specific signatures of white matter pathology in tauopathies
Source: Acta Neuropathol Commun. 2021 Oct 21;9:170. doi: 10.1186/s40478-021-01271-x (PMC8529809; doi:10.1186/s40478-021-01271-x)
Supplement: Supplementary file 1 — Additional file 1. This file contains supplementary figures S1–S8 and tables S1–S4 describing data and additional analyses related to the study. [file 40478_2021_1271_MOESM1_ESM.pdf]

Supplemental Fig. S1 Multiple Instance Learning Model Architecture. Diagram depicting the architecture of our custom multiple instance learning slide model.

Supplemental Fig. S2 Disease-specific Performance of Region Classifier. Similar to Fig.2a, except performance is broken down on a per-disease basis. **a** Confusion matrices showing average region identification accuracy for each disease. **b** Extended examples of classification results overlaid on WSIs (cortex: cyan, white matter: magenta, background: no color).

Supplemental Fig. S3 Disease-specific Performance of Aggregate Classifier. Similar to Fig.2b, except performance is broken down on a per-disease basis. **a** Confusion matrices showing average aggregate identification accuracy for each disease. **b** Confusion matrix showing average aggregate identification accuracy for data from cortex regions (left) and example of aggregate-dense regions in the cortex (right).

Supplemental Fig. S4 Performance of Aggregate Identification Model. Comparison of ground truth and predicted aggregate features from each WSI. For all annotated regions from a WSI, we extracted nine handcrafted features from both annotated aggregates and aggregates predicted by our model. We then plotted in a scatter plot the slide median value for each feature from these two groups and calculated the Spearman Rank Correlation. Red line indicates ideal prediction with X=Y. Note: All plots used the same number of slides, however as features such as Width and Extent were measured in discrete pixel units, multiple slides displayed exactly the same median values and appear as a single point.

Supplemental Fig. S5 Detection and Removal of Aggregate Artifacts. **a** 200 aggregates were randomly subsampled from each WSI and UMAP Clustering of aggregates (individual points colored by disease) was performed using all hand-crafted features including texture features; cluster of aggregate artifacts are circled and example aggregate and artifact patches are shown. **b** Examples of aggregate objects classified as an artifact by a trained random forest classifier. **c** Examples of aggregate objects classified as a true aggregate by a trained random forest classifier. **d** Hierarchical clustering of WSIs using their average aggregate feature values before artifact removal.

Supplemental Fig. S6 Outliers from hierarchical clustering (Fig. 4a) exhibit low tau burden in the WM. **a** Six AD WSIs show low tau burden distinct from other AD samples, while a single CBD outlier based on aggregate shape features also exhibits low tau burden. **b** Corresponding data from hierarchical clusters are indicated by colored arrows.

Supplemental Fig. S7 Distribution of slide median values for all hand-crafted features. Similar to 4c, except all features used in Fig.4a are shown. For each handcrafted feature used in analysis, boxplots are shown comparing the slide-average values between diseases. Mann-Whitney test, with Bonferroni-based multiple-hypothesis testing correction (across diseases) was used for statistical comparison.

Supplemental Fig. S8 WM and CTX models are complementary **a** Slide-level performance comparison of cortex/WM models. Disease classification accuracy on a WSI based on WM (x-axis) and cortex (y-axis) models is separately binned into 4 levels (0-25%,25-50%,50-75%,75-100% of patches assigned to correct disease) and the number of WSI in each bin is depicted. **b** Stacked bar plot showing the number and disease type of WSIs misclassified (i.e., whether the class most patches were assigned to was not the correct one) by either model separately or by the consensus prediction of both models.

Supplemental Fig. S1 MIL Architecture

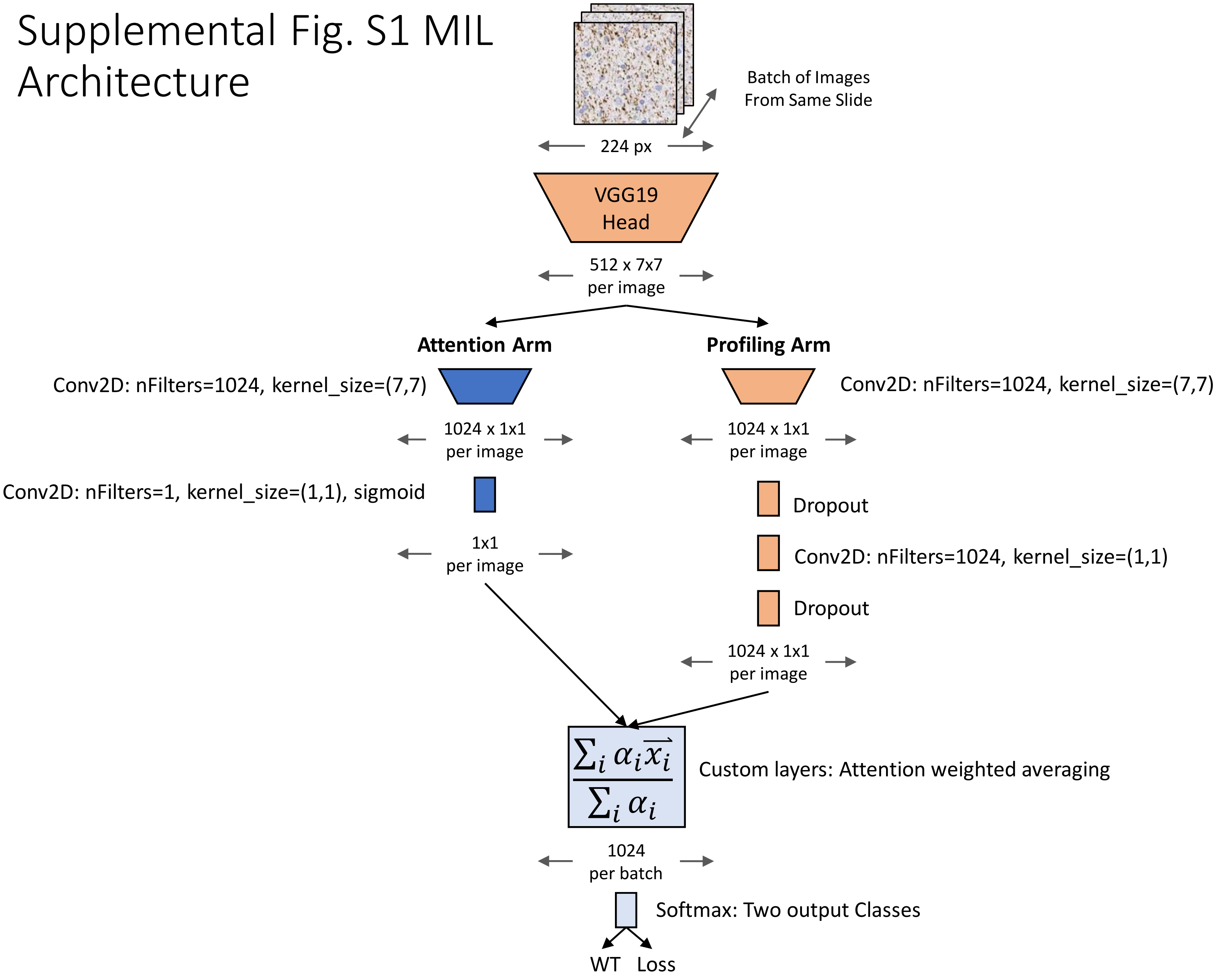

Supplemental Fig. S2 Disease Specific Performance of Region Classifier

a.

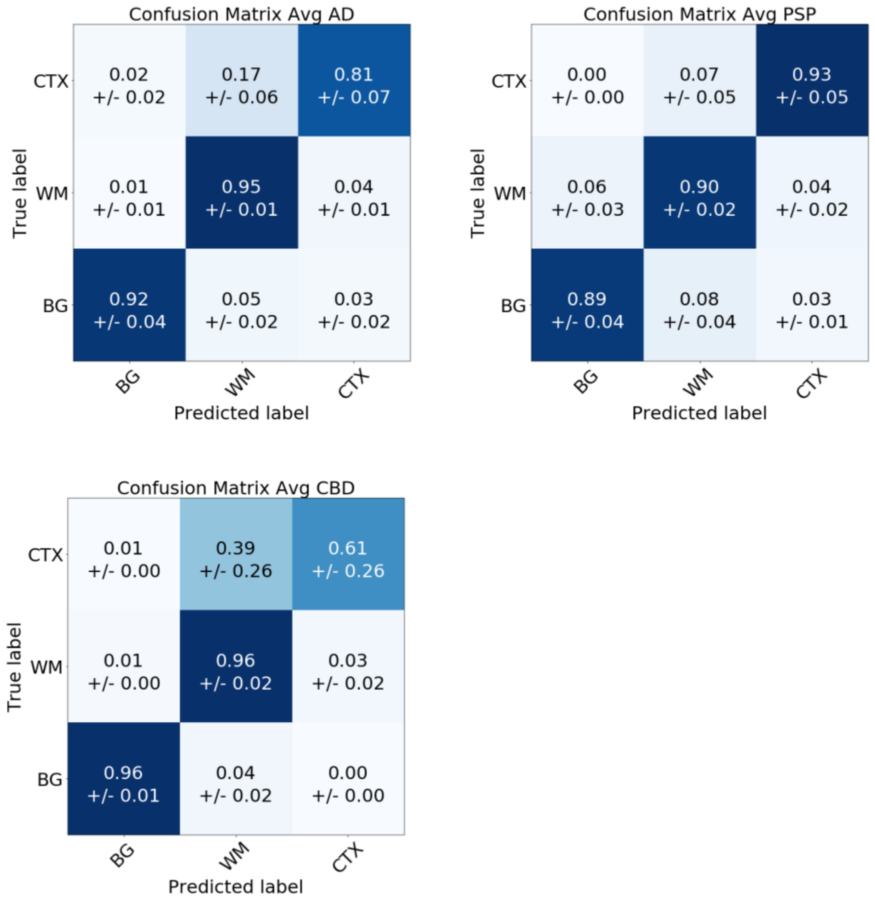

b.

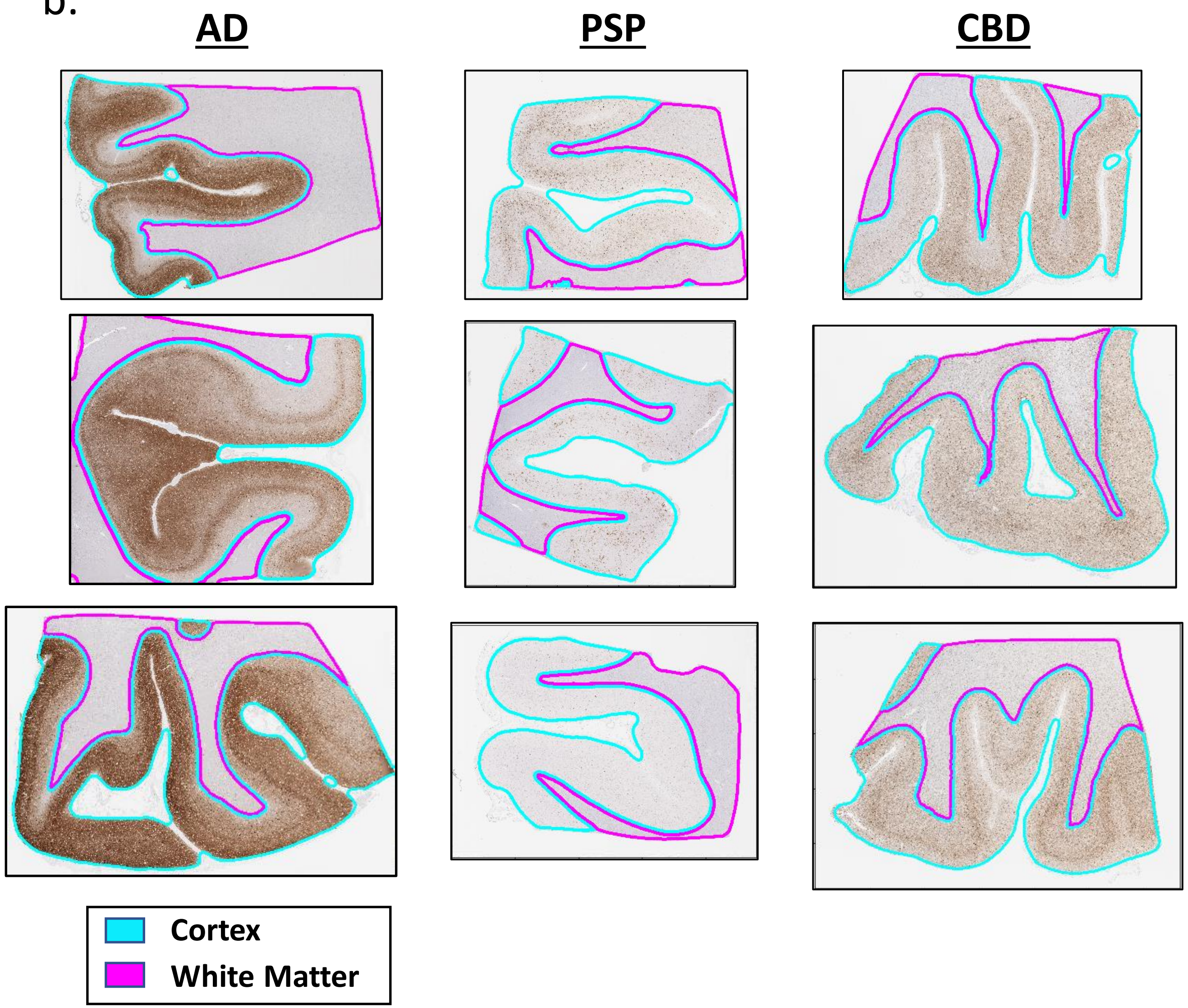

Supplemental Fig. S3 Disease Specific Performance of Aggregate Classifier

a.

White Matter

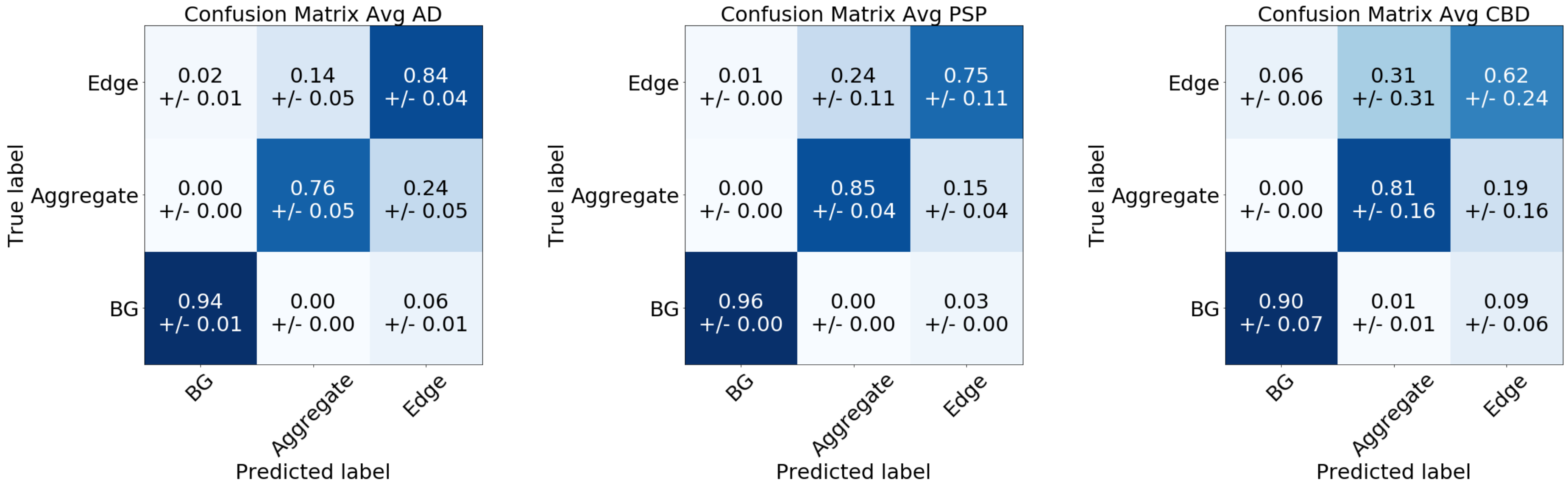

b.

Cortex

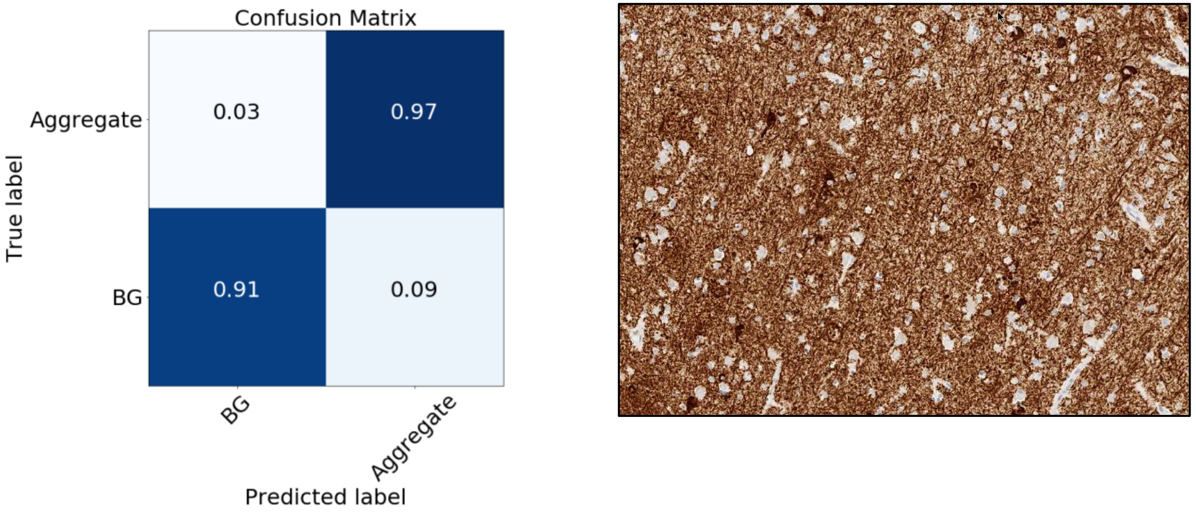

Supplemental Fig. S4 Performance of Aggregate Identification Model

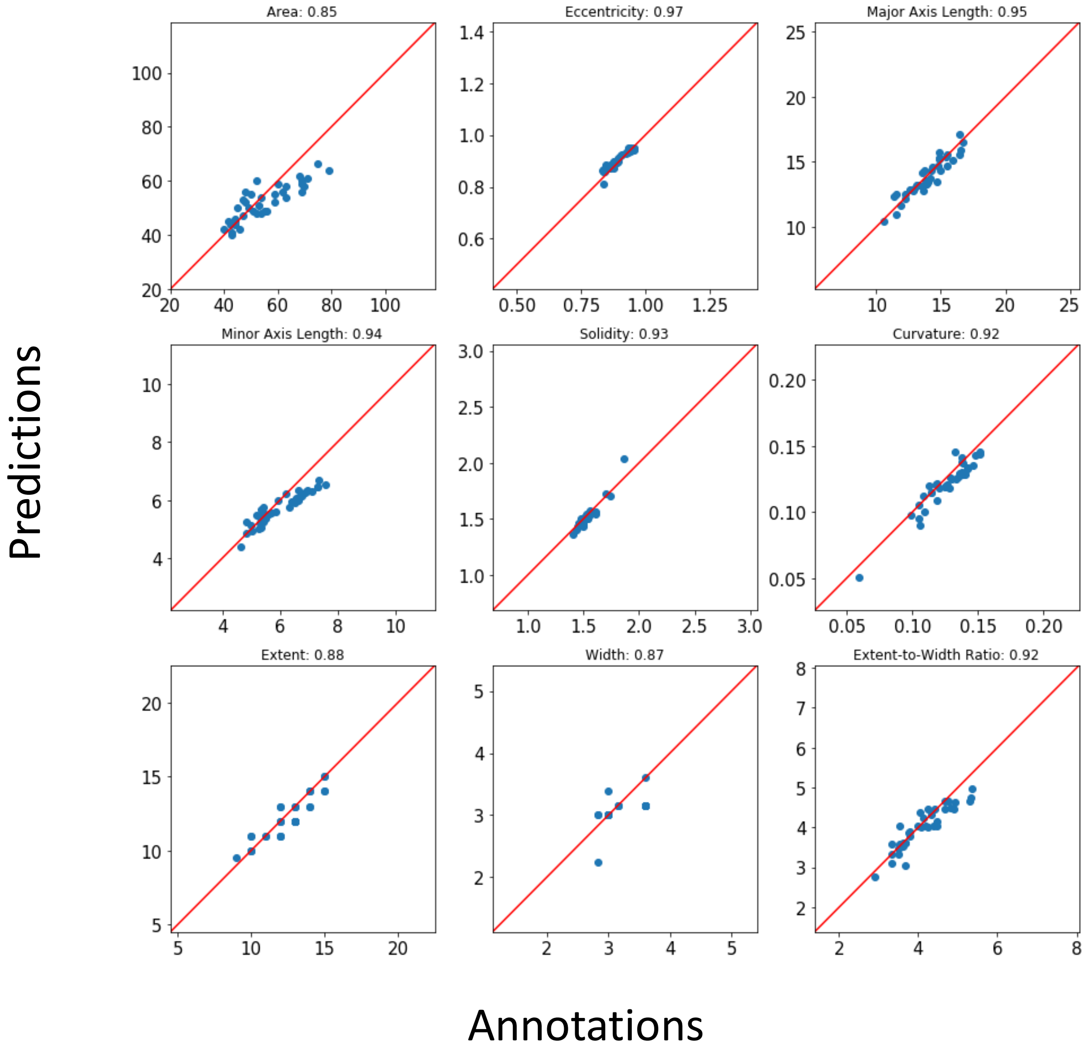

Supplemental Fig. S5 Detection and removal of aggregate artifacts

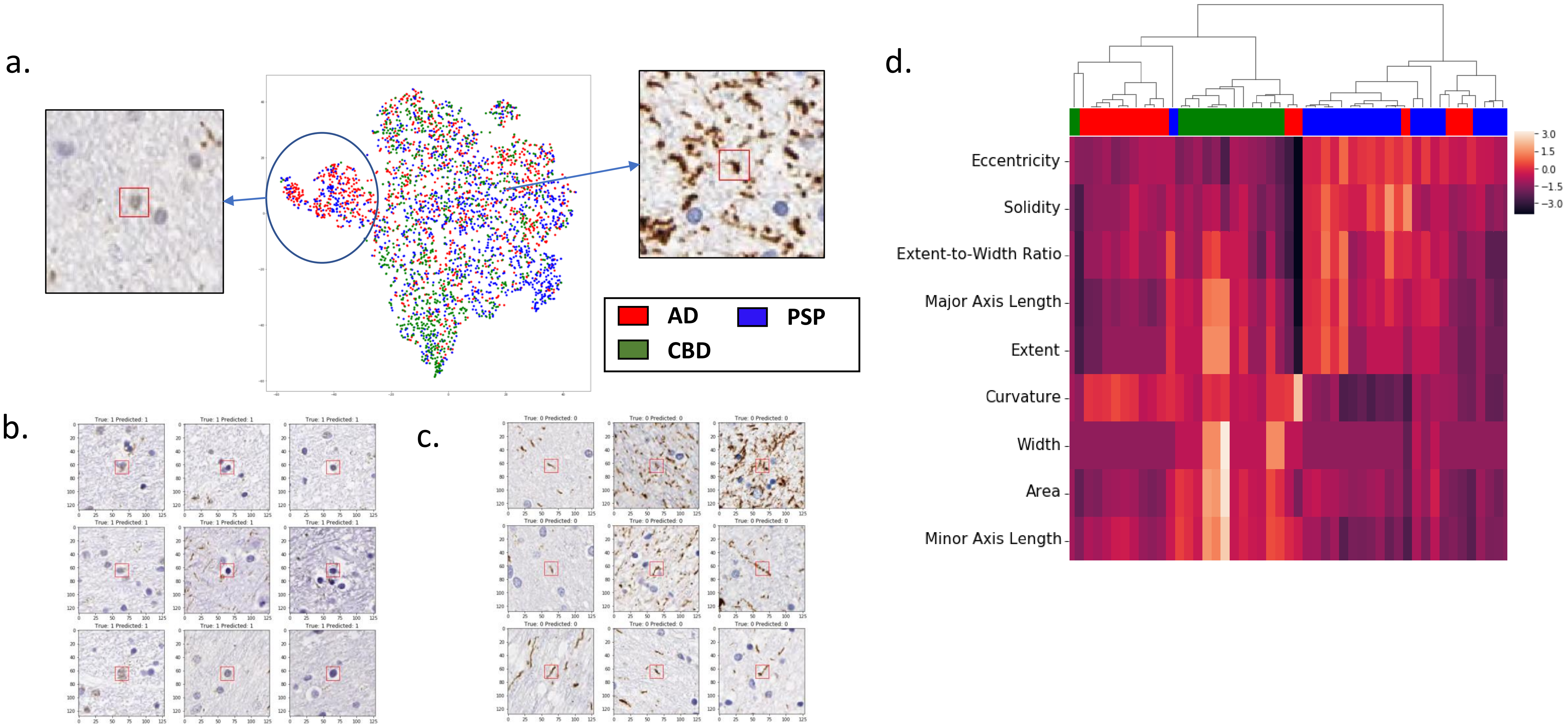

Supplemental Fig. S6 Outliers from hierarchical clustering exhibit low tau burden in the WM

a. Tau Burden Outliers using Log scale

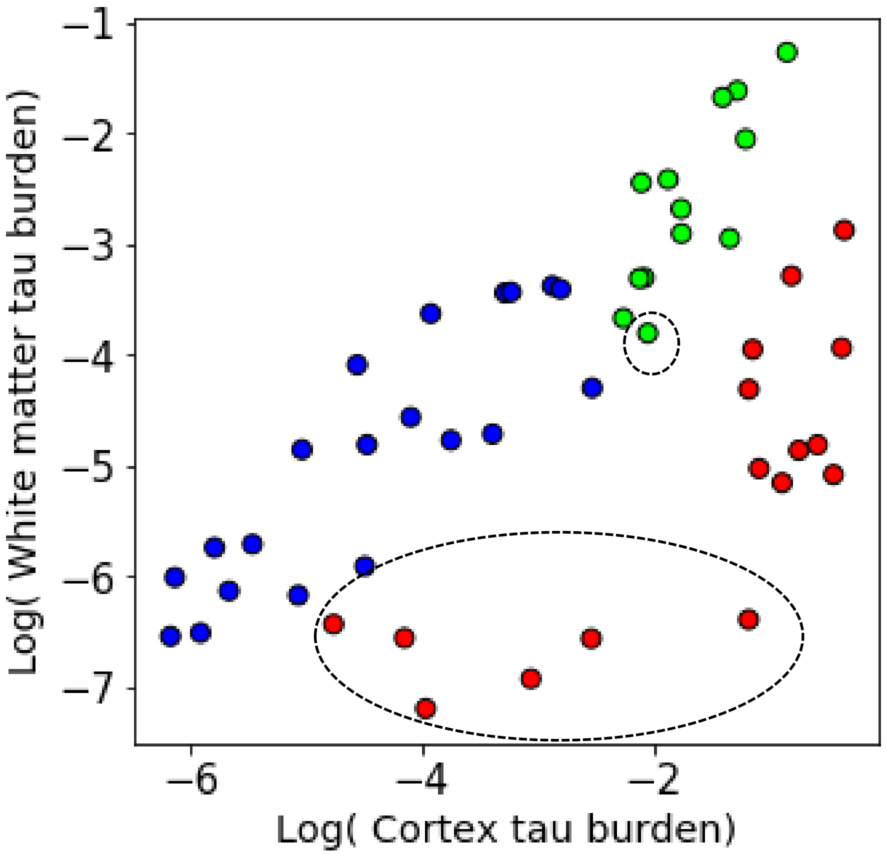

b. Outlier Location in Hierarchical Cluster

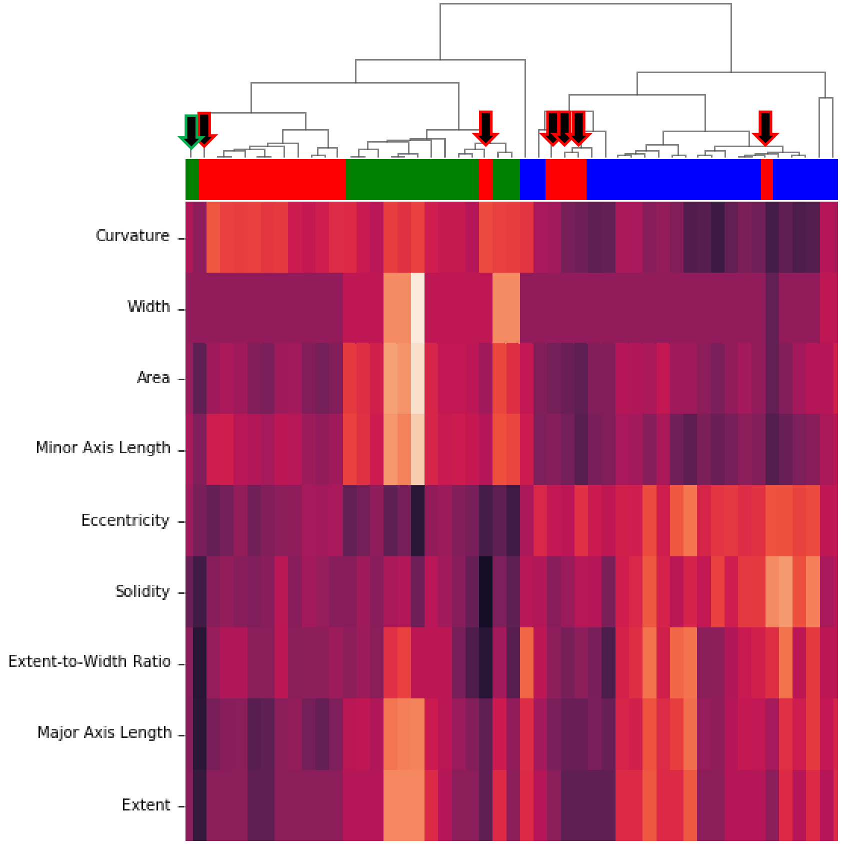

Supplemental Fig. S7 Distribution of slide average values for all hand-crafted features

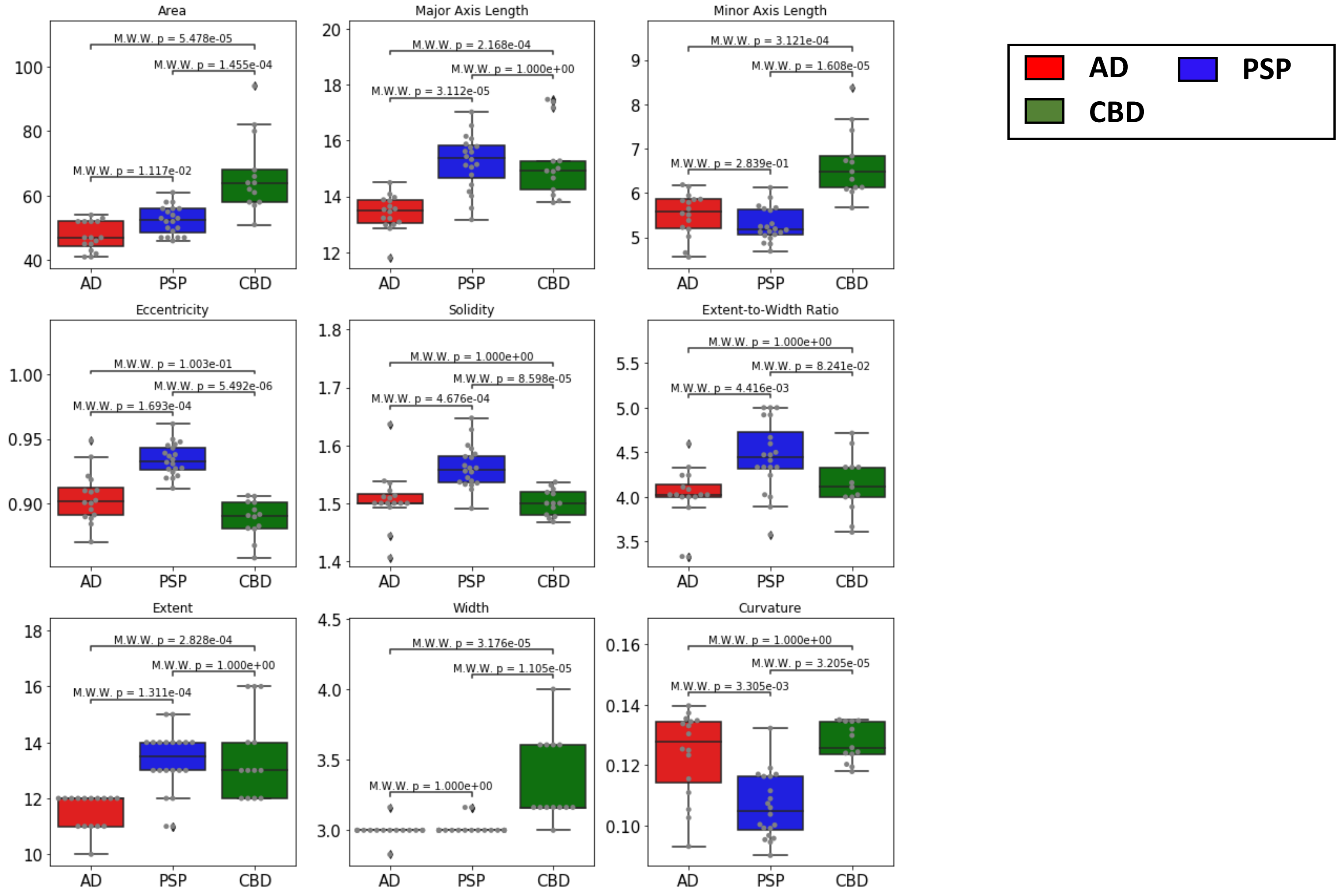

Supplemental Fig. S8 WM and CTX models are complementary

a. **Slide-level Performance Comparison**

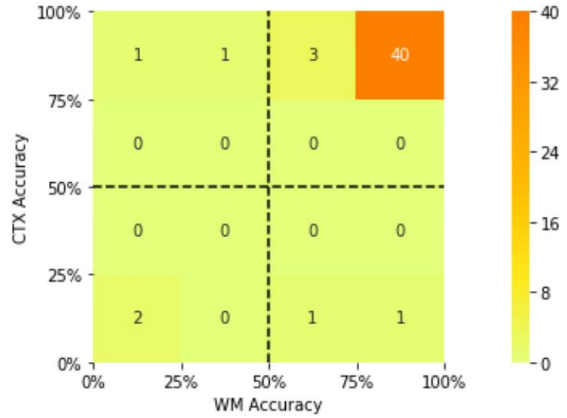

b. **Types of Slide Misclassifications**

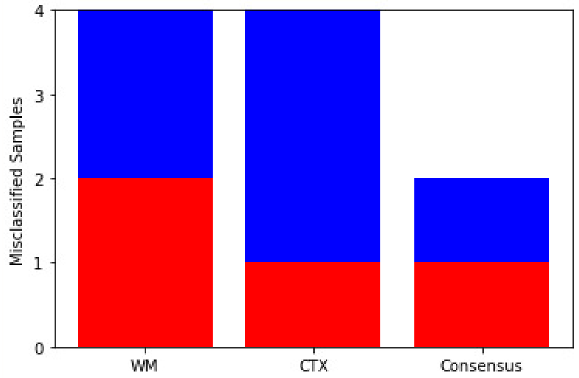

Supplementary Table S1. Demographic Characteristics

	AD	PSP	CBD
Age			
Median	75.5	75	73
Range	56-91	59-91	55-86
Sex			
Male (n)	10	9	10
Female (n)	6	11	3
Ratio	1.7:1	1:1.2	3.3:1
Total (n)	16	19	13

Supplementary Table S2. Region Classification Network Architecture

Layer (type)	# Filters	# Parameters
Conv2D	64	3,136
MaxPooling2D		
Conv2D	32	32,800
MaxPooling2D		
Conv2D	32	16,416
MaxPooling2D		
Conv2D	32	36,896
Conv2D	32	6,147
Activation (Softmax with 3 outputs)		
Flatten		

Supplementary Table S3. Handcrafted Features

	Feature	Description
1	Area	Total number of pixels within aggregate object
2	Curvature	Aggregates were first skeletonized, and local curvature was calculated as the reciprocal of the radius of the best fitting circle. This curvature was then averaged across the aggregate.
3	Extent	Geodesic distance in pixels between the two furthest points in an aggregate object
4	Extent-Width Ratio	Ratio of the measured extent and width for an aggregate object
5	Eccentricity	Ratio of the minor and major axis length of an aggregate object
6	Major Axis Length	Length in pixels of the major axis of an ellipse that has the same normalized central moments as the aggregate object
7	Minor Axis Length	Length in pixels of the minor axis of an ellipse that has the same normalized central moments as the aggregate object
8	Width	Distance in pixels from the most central point of an aggregate object to the edge of the object
9	Solidity	Proportion of pixels within the convex hull of the aggregate object that are also within the aggregate object (i.e. Area/ Convex Area)

Supplementary Table S4. Texture Features

	Feature	Description
1	Haralick 2nd Moment	Haralick feature 1 (Angular 2nd moment) of AT8 intensity across all pixels in the aggregate
2	Haralick Correlation	Haralick feature 3 (Correlation) of AT8 intensity across all pixels in the aggregate
3	Haralick Sum Average	Haralick feature 6 (Sum Average) of AT8 intensity across all pixels in the aggregate
